# Suicide attempts in patients with acute and transient psychotic disorders in in-patient centers in Latvia 2014-2019

**DOI:** 10.1192/j.eurpsy.2022.2167

**Published:** 2022-09-01

**Authors:** I. Germanenko, J. Vrubleska, E. Rancans

**Affiliations:** Riga Stradins University, Department Of Psychiatry And Narcology, Riga, Latvia

**Keywords:** Suicidology, PSYCHOTIC DISORDERS, Epidemiology

## Abstract

**Introduction:**

Acute and transient psychotic disorders (ATPDs) are a group of diagnoses with acute onset and polymorphic psychotic symptoms, divided into 6 subtypes, which may or may not associate with acute stress. Suicide is a serious public health problem, having long-lasting effects on social well-being and economics. The prevalence of suicide attempts (SA) in Latvia remains unknown, several studies are currently in progress.

**Objectives:**

To determine the prevalence of SA among patients with ATPDs and features of this subgroup of patients.

**Methods:**

Retrospective study based on socio-demographic data and additional diagnoses of all patients with ATPDs provided by the National Center of Statistics of Diseases.

**Results:**

There were 1779 patients with ATDPs in 2014-2019, 44 people were admitted with a SA (24 men, 54.5%). There was a significant difference between sexes in the mean age – 39.64±14.66 for women, 31.94±11.88 for men (p<.001), as well as in case of associated acute stress – 37.61±12.95 with and 35.33±13.15 without it (p=.01). There were no differences in age or ATPDs subtypes between patients with SA and without one. Men with SA abused alcohol in everyday life more than women and men without SA (20%,p<.001), women with SA had acculturational difficulties more often than men and women without SA (45%,p<.001). Intentional self-harm by sharp objects was more common among men (p<.001), intentional self-poisoning among women (p<.001).

**Conclusions:**

The prevalence of SA among patients with ATPDs was 2.47%. Factors, which were more commonly observed in patients with SA were alcohol consumption and acculturational difficulties. Types of self-harming differ between sexes.

**Disclosure:**

No significant relationships.

